# Antagonistic Roles for H3K36me3 and H3K27me3 in the Cold-Induced Epigenetic Switch at *Arabidopsis FLC*

**DOI:** 10.1016/j.cub.2014.06.047

**Published:** 2014-08-04

**Authors:** Hongchun Yang, Martin Howard, Caroline Dean

**Affiliations:** 1 Department of Cell and Developmental Biology, John Innes Centre, Norwich Research Park, Norwich NR4 7UH, UK; 2 Computational and Systems Biology, John Innes Centre, Norwich Research Park, Norwich NR4 7UH, UK

## Abstract

Posttranslational modifications of histone tails are an important factor regulating chromatin structure and gene expression. Epigenetic memory systems have been predicted to involve mutually exclusive histone modifications that, through positive feedback mechanisms, generate bistable states [1, 2]. How the states are interconverted is not understood, and whether the histone modifications are sufficient for epigenetic memory is still greatly debated [3]. We have exploited the process of vernalization, the slow quantitative epigenetic silencing of *Arabidopsis FLC* induced by cold, to detail with fine temporal and spatial resolution the dynamics of histone modifications during an epigenetic switch. The profiles of H3K36me3, H3K4me3, and H3K4me2 at *FLC* throughout the vernalization process were compared to H3K27me3, which accumulates at a local nucleation region during the cold and spreads across the locus on return to the warm [2]. We find for many phases of the vernalization process that H3K36me3 and H3K27me3 show opposing profiles in the *FLC* nucleation region and gene body, that H3K36me3 and H3K27me3 rarely coexist on the same histone tail, and that this antagonism is functionally important. A lack of H3K36me3 results in a fully silenced state at *FLC* even in the absence of cold. We therefore propose that H3K36me3 is the opposing modification to H3K27me3 in the Polycomb-mediated silencing of *FLC*. However, the lack of an absolute mirror profile predicted from modeling suggests that their antagonistic roles contribute a necessary, but not sufficient, component of the mechanism enabling switching between and inheritance of epigenetic states.

## Highlights

• Modeling suggests that epigenetic systems involve bistable states
• These bistable states involve mutually exclusive histone modifications
• H3K36me3 and H3K27me3 appear to be opposing modifications at *FLC*
• H3K36me3/H3K27me3 balance at *FLC* “registers” cold exposure

## Teaser

Yang et al. investigate histone modification dynamics during the cold-induced epigenetic switching that occurs at the *FLC* locus during vernalization. H3K36me3 shows an opposing profile to H3K27me3 at the nucleation region, and Yang et al. propose that the dynamic balance between these two modifications is a register of cold exposure.

## Results and Discussion

Previously, we developed a mathematical model aimed at explaining the quantitative basis of the epigenetic silencing of *FLC* during vernalization [2]. The model incorporates a highly dynamic chromatin environment in which opposing histone modifications are constantly being added and removed. Through implicit recruitment of appropriate protein complexes, one type of histone modification can promote both the addition of further modifications of the same type and removal of modifications of the opposing type. Such positive-feedback mechanisms can result in either of the antagonistic histone modifications becoming self-sustaining, leading to opposing epigenetically stable states [1, 2, 4, 5]. For *FLC*, we envisioned an actively transcribed “A” state that switches to a stably repressed “M” state during vernalization. Genetic and chromatin immunoprecipitation (ChIP) analysis had shown that Polycomb activity, which induces H3K27me3 modifications, was consistent with the M state at *FLC* [2, 6–8]. The targeted nucleation of a modified Polycomb complex—PHD-PRC2—at a localized “nucleation” region within *FLC*, enabled switching of *FLC* to an epigenetically stable silent expression state [2, 6–9]. This switch was associated with spreading of the PHD-PRC2 complex and enhanced H3K27me3 levels across the body of the gene when the plants were returned to warm conditions [9]. The model predicted that the quantitative nature of vernalization would be achieved through a population average, with longer exposure to cold leading to a higher proportion of cells in which *FLC* loci have digitally switched to the fully silenced M state. This prediction was validated through analysis of an FLC-GUS fusion in *Arabidopsis* plants [2].

**Figure 1:**
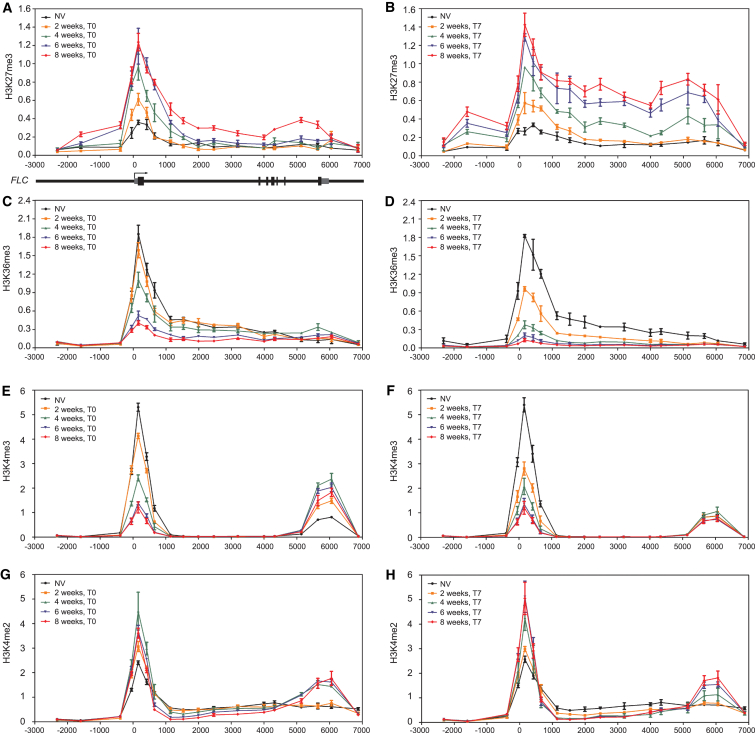
Histone Modifications at *FLC* during Vernalization (A, C, E, and G) H3K27me3 (A), H3K36me3 (C), H3K4me3 (E), and H3K4me2 (G) profiles across the *FLC* locus for nonvernalized plants (NV) and after different lengths (2, 4, 6, and 8 weeks) of cold treatment, without postcold growth (T0). *FLC* gene structure is shown schematically at the bottom of (A). (B, D, F, and H) H3K27me3 (B), H3K36me3 (D), H3K4me3 (F), and H3K4me2 (H) profiles across *FLC* locus for nonvernalized plants and after different lengths (2, 4, 6, and 8 weeks) of cold treatment, with 7 days postcold growth (T7). Data were presented as the ratio of (*FLC*/H3) to (reference gene/H3). *STM* was used as the reference gene for H3K27me3 and *ACTIN* was used for H3K36me3, H3K4me3 and H3K4me2. Values represent the average and SEM of three independent biological replicates in all cases. See also Figure S1 and Table S1.

A further, fundamental prediction of the model was the existence of an opposing state to the stably repressed M state. This opposing A state would be likely to involve a histone modification associated with active gene expression—in *S. pombe*, the opposing modifications were thought to be acetylation/methylation of the same residue on the histone tail [4]. We therefore systematically screened the dynamic behavior of a series of histone modifications at *FLC* before, during, and after cold exposure and compared them with the H3K27me3 profile. Higher-resolution ChIP analysis (compared to our previous analysis) was used on material showing quantitative epigenetic silencing of *FLC* expression (Figure S1A available online) [2, 10]. A basal level of H3K27me3 was found across *FLC*, consistent with the low levels of PRC2 before cold exposure [9], with a slight peak at the nucleation region just downstream of the transcription start site (TSS) (Figure 1A and Table S1). During the cold, H3K27me3 levels accumulated quantitatively at the nucleation region, saturating after 6 weeks of cold (Figure 1A), consistent with the PHD-PRC2 complex accumulating at the same region during cold exposure [9]. At 8 weeks of cold exposure, the H3K27me3 began to spread slightly across the locus, but this spreading predominantly occurred after plants were transferred back to warm conditions for 7 days [11] (Figure 1B).

The model predicts that the A state modification should show a mirror image of the H3K27me3 profile at *FLC*, with high levels before vernalization across the whole *FLC* gene [2]. Genome-wide analysis in many organisms has suggested H3K36me3 and H3K36me2 generally accumulate to high levels in gene bodies through linkage with productive transcription [12–14]. We therefore analyzed the profiles of H3K36me2 and H3K36me3 at *FLC*. H3K36me2 accumulation was very low before cold, comparable to the negative control (Figure S1B), and did not obviously change in plants given cold treatment (Figure S1C). However, the H3K36me3 profile across *FLC* was rather different, with a strong peak at the nucleation region and somewhat elevated levels across the gene body before cold (Figure 1C). The H3K36me3 accumulation at the nucleation region was gradually suppressed with increasing cold. The reduction between 2 and 6 weeks of cold was strongest and coincided with the largest increases in H3K27me3 in the nucleation region. H3K36me3 outside the nucleation region also decreased, such that after 6 weeks of cold H3K36me3 levels in the gene body were very low (Figure 1C). The H3K36me3 profile that we observed at *FLC* was distinct from some that of other organisms, and we also found similar patterns at two housekeeping genes, *ACTIN* and *UBC* (Figures S1D and S1E). Consistently, a metagene analysis of the *A. thaliana* genome has also shown an H3K36me3 profile over genic regions that differs from that seen in yeast and mammals [15], so H3K36me3 may play a different role in the *Arabidopsis* genome. Overall, there was a significant anticorrelation between H3K36me3 and H3K27me3 at all locations across *FLC* during the different phases of the vernalization process. The antagonism between H3K36me3 and H3K27me3 was, however, clearest at the nucleation region of *FLC* as the plants experienced an increasing duration of cold. The pattern of decreasing H3K36me3 levels as H3K27me3 increased (Figures 1A–1D) fits the opposing pattern expected from the model. However, the relatively low H3K36me3 levels in the gene body before vernalization did not fit predictions of the model—the opposing modification was expected to be at higher levels over the gene body in order to ensure mitotic stability through many generations of cell division.

H3K4me3 and H3K4me2 are two other well-characterized histone modifications associated with gene activation [16, 17]. These two modifications have previously been shown to be important for *FLC* regulation [18–20]. Therefore, we analyzed the dynamics of H3K4me3 and H3K4me2 localization in the different regions of *FLC* at different phases of vernalization. Before cold, H3K4me3 was enriched over just two regions of *FLC*, a high peak at the nucleation region and a smaller peak at the 3′ end of sense *FLC* (distal peak) (Figure 1E). The position of the latter peak corresponds to the promoter of a set of *FLC* antisense transcripts called *COOLAIR* [21]. Very little other H3K4me3 enrichment was found in the *FLC* gene body (from +1,000 to +5,000) (Figure 1E). During cold exposure, H3K4me3 levels in the nucleation region gradually decreased, reaching their lowest level after 6–8 weeks of cold (Figure 1E). As was observed for H3K36me3, H3K4me3 levels in plants given 2 and 4 weeks of cold continued to drop when the plants were transferred to the warm (Figures 1E and 1F). Interestingly, the 3′ H3K4me3 peak gradually increased for the first 4 weeks of cold, then slowly came down (after 6 and 8 weeks of cold) (Figure 1E). This coincides with the increase in *COOLAIR* transcription during the first few weeks of cold exposure [21]. After transfer back to the warm, the distal peak of H3K4me3 returned to precold levels (Figure 1F), again coinciding with low levels of *COOLAIR* transcription. Thus, H3K4me3 and H3K27me3 dynamics showed opposing dynamics at the nucleation region, but this did not extend to the gene body, where H3K4me3 levels were extremely low, or to the 3′ end of *FLC*, over the *COOLAIR* promoter. Hence, H3K4me3 does not have the characteristics that suggest that it functions as an opposing mark to H3K27me3 at *FLC*. In mouse and human embryonic stem cells, a large number of PRC2 targets were covered by bivalent domains with H3K4me3 and H3K27me3, but few were modified by H3K36me3 [22, 23]. This implies that, in general, H3K4me3 is unlikely to be the opposing mark to H3K27me3.

The dynamics of H3K4me2 were also measured over *FLC* during vernalization. Before cold exposure, H3K4me2 was enriched over the whole locus, with the highest levels at the nucleation region and lowest levels in the gene body and 3′ end of *FLC* (Figure 1G). During the cold, H3K4me2 dynamics showed distinct behaviors in the different *FLC* regions. At the nucleation region, H3K4me2 accumulated for the first 4 weeks of cold, and then decreased slightly. Within the gene body, H3K4me2 gradually decreased during the cold and a small 3′ peak developed over the *COOLAIR* promoter, but only after 4 weeks, and then did not increase any further (Figure 1G). The increased level of H3K4me2 at the nucleation region after 2 and 4 weeks of cold was stable after transfer back to the warm. However, after return to the warm after 6 and 8 weeks of cold exposure, H3K4me2 levels increased further. In the *FLC* gene body, there was a small reduction of H3K4me2 after return to the warm, with the largest peak in the 3′ region at the *COOLAIR* promoter, where we observed different dynamics as compared to H3K4me3 (Figure 1H). Overall, these dynamics show that H3K4me2 is also unlikely to act as the opposing modification to H3K27me3 during the switch of expression states in vernalization.

**Figure 2:**
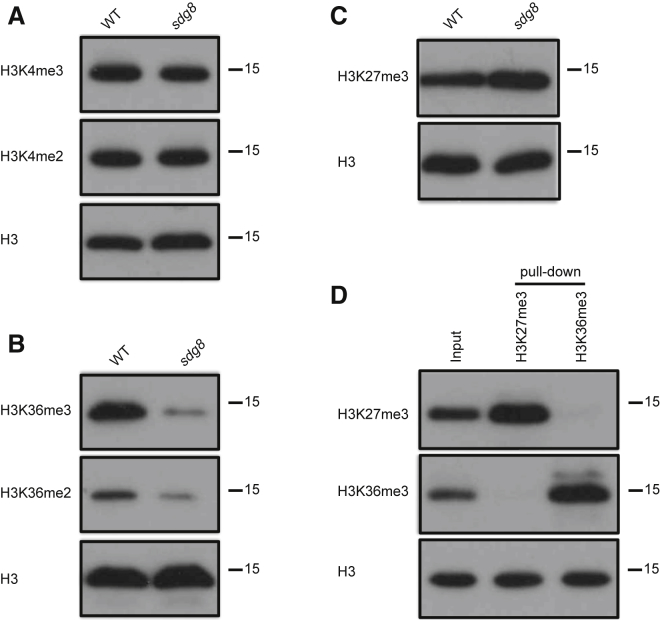
H3K36me3 Is Mainly Delivered by *SDG8* and Rarely Coexists with H3K27me3 on the Same Histone Tail (A) Global levels of H3K4me3 and H3K4me2 in the wild-type (WT) and *sdg8* mutant. Antibodies used are shown on the left. Molecular weights (kDa) are indicated on the right. Histone H3 was used as the loading control. (B) Global levels of H3K36me3 and H3K36me2 in the WT and *sdg8* mutant. (C) Global level of H3K27me3 in the WT and *sdg8* mutant. (D) H3K36me3 and H3K27me3 rarely coexist on the same histone tail, as shown by immunoprecipitation and western blotting. Histone H3 was used as the loading control.

None of the H3K36me3, H3K36me2, H3K4me3, and H3K4me2 modifications analyzed exactly fitted the theoretically predicted pattern for the opposing modification to H3K27me3 at *FLC* during all phases of the vernalization process. However, H3K36me3 fitted most closely over both the nucleation region and gene body, although levels over the gene body were lower than predicted prior to the cold. To further verify the predicted functional antagonism between H3K36me3 and H3K27me3, we assayed H3K27me3 levels in genotypes deficient in H3K36me3. SDG8 has recently been shown to be the specific methyltransferase for H3K36 at *FLC* [24], despite previous confusion over whether SDG8 methylated H3K36 and H3K4 [25–28]. We extended this finding to show that global levels of H3K4me3 and H3K4me2 were unchanged in the *Arabidopsis sdg8* mutant (Figure 2A), but H3K36me3 and H3K36me2 decreased significantly (Figure 2B). Our data therefore implicate SDG8 as the major H3K36me3 methyltransferase in the *Arabidopsis thaliana* genome. Importantly, western blots suggest that global levels of H3K27me3 are also increased in *sdg8* (Figure 2C), suggesting that H3K36 methylation antagonizes H3K27me3 in a genome-wide manner.

**Figure 3:**
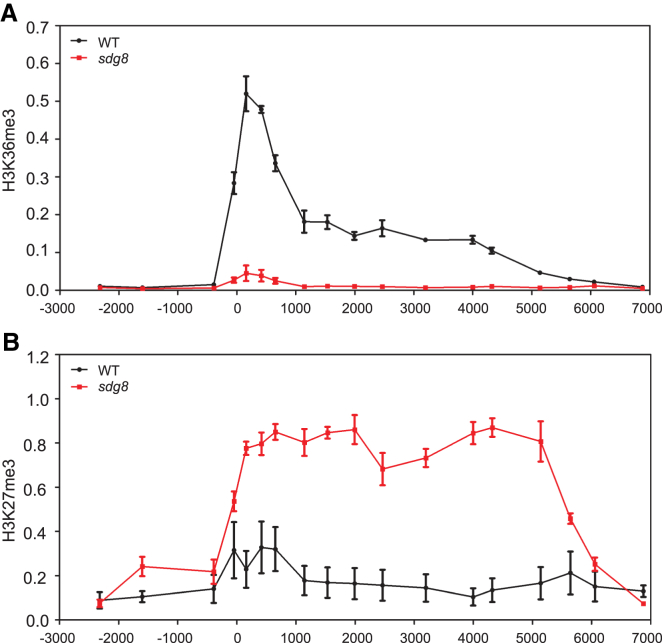
*FLC* Is in a Fully Silenced State in the *sdg8* Mutant (A) H3K36me3 levels at *FLC* in an *sdg8* mutant relative to H3 (H3K36me3 *FLC*/H3 *FLC*). (B) H3K27me3 pattern at *FLC* in an *sdg8* mutant. Data are shown as the ratio of H3K27me3 *FLC*/H3 *FLC* to H3K27me3 *STM*/H3 *STM*. Seedlings were harvested in nonvernalized conditions. Values represent the average and SEM of three independent biological replicates. See also Figure S2.

Considering this antagonism between H3K36me3 and H3K27me3, we asked whether the two modifications could colocalize on the same histone tail in *Arabidopsis thaliana*. Analysis of nucleosomes in mouse embryonic stem cells, mouse embryonic fibroblasts, and HeLa cells has shown H3K27me2/H3K27me3 occurs both asymmetrically and symmetrically, i.e., on one or both histone tails of each nucleosome [29, 30]. Bivalent nucleosomes carry H3K4me3 or H3K36me3 along with H3K27me3 on opposite H3 tails and resolve to either one or other modification states during differentiation [29]. We extracted histones from disrupted nucleosomes and enriched the H3K27me3 fraction using an anti-H3K27me3 antibody. Strikingly, H3K36me3 was hardly detected in the enriched H3K27me3 histone fraction (Figure 2D) [31]. Conversely, H3K27me3 was excluded from an enriched H3K36me3 histone fraction (Figure 2D). These data show H3K36me3 and H3K27me3 rarely coexist on the same histone H3 tail, which is consistent with the model prediction of exclusive M and A modifications. We then focused on the antagonistic dynamics of H3K36me3 and H3K27me3 at *FLC*. We measured H3K36me3 and H3K27me3 levels at *FLC* in an *sdg8* mutant. As global levels of H3K36me3 are affected by *sdg8*, it is difficult to find a good reference gene for the ChIP experiments (Figure S2A), so we normalized all H3K36me3 data to H3 directly. For H3K27me3, no significant difference was found at *STM* between the wild-type and the *sdg8* mutant (Figure S2B), so *STM* was used as the reference gene. Consistent with the large reduction in H3K36me3 globally, we found a large decrease in H3K36me3 at *FLC* in *sdg8* (Figure 3A) and a correspondingly large increase in H3K27me3 (Figure 3B), similar in extent to the fully vernalized state (seen at 8 weeks of cold followed by 7 days of warm; Figure 1B). *FLC* expression analysis shows that *FLC* is fully silenced in *sdg8* (Figure S2C), so loss of an H3K36 methyltransferase leads to a reduction of H3K36me3 at *FLC* and upregulation of H3K27me3. This finding is again in agreement with our earlier modeling, in which loss of the activating A mark (H3K36me3) would lead to M modifications (H3K27me3) spreading across the entire locus, with associated epigenetic silencing.

The high-resolution temporal and spatial analysis shown here has now provided a clear picture of the dynamics of a series of histone modifications at the *FLC* locus during the phases of vernalization. The intent was to discover a modification with a pattern that was a mirror to that of H3K27me3, known to be centrally important in the Polycomb-induced silencing mechanism [2]. None of the profiles of the active histone modifications exactly fitted the mirror pattern for the opposing epigenetic state predicted from earlier modeling. However, similar to model predictions, a clear antagonism was discovered between H3K36me3 and H3K27me3, both for the nucleation region and the gene body. In particular, the two modifications were unable to coexist on the same histone H3 tail, and suppression of H3K36me3 (*sdg8* mutant) led to H3K27me3 upregulation. Furthermore, in the nucleation region, the quantitative decrease in H3K36me3 and mirror-like quantitative increase in H3K27me3 suggest that they are indeed markers of opposing states that switch with increasing cold exposure.

However, outside of the nucleation region, a mirror-like opposition of states between H3K36me3 and H3K27me3 applied less well. Importantly, H3K36me3 was only present at relatively low levels across the gene body prior to cold exposure, in contrast to the high levels predicted by modeling. Potentially, epigenetic memory could be written only in the nucleation region, where mirror H3K36me3/H3K27me3 states do exist. However, this interpretation is problematic due to the relatively small number of nucleosomes (approximately three, so approximately six histone 3 molecules) in the nucleation region. Complete loss of epigenetic marks in this limited region, either at DNA replication or simply through nucleosome swap-outs, is likely to occur with comparatively high frequency, leading to epigenetic memory loss. Hence, it is unlikely that a purely histone-based feedback mechanism could confer epigenetic memory between cell divisions based only on this limited region. Moreover, a similar conclusion is reached on consideration of the lack of a mirror image of the modifications from the low levels of H3K36me3 outside the nucleation region before cold. Accordingly, our earlier models of the vernalization process will need to be updated to reflect this analysis, with H3K36me3/H3K27me3 dynamics being necessary, but not sufficient, for epigenetic switching and memory. Additional mechanisms, potentially involving *trans*-factors, noncoding RNAs, other histone modifications, or proteins that remain bound to DNA through its replication, could encode the robust positive feedback required to enforce epigenetic inheritance of active or repressed states [1].

The dynamics during cold exposure at *FLC* and the genome-wide antagonism between H3K36me3 and H3K27me3 suggest a close functional connection between the complexes delivering H3K36me3 and H3K27me3. The activity of H3K36 methyltransferase has been shown to inhibit the enzyme activity of the PRC2 H3K27me3 complex [29, 30, 32]. This mutual inhibition is again similar to interactions predicted from our earlier modeling, in which each histone mark was assumed to recruit factors that not only reinforced the presence of that mark, but that also inhibited the opposing mark. Moreover, a similar feature extends to the PRC2 complex, which also contains associated proteins that recognize H3K36me3 and recruit H3K36 demethylase to facilitate its activity [23, 33–35].

An interesting open question for the future is whether the gradual change from H3K36me3 to H3K27me3 observed at the nucleation region during cold exposure involves progressive, gradual changes in modification levels of the different histone tails in all cells. An alternative to this “analog” mechanism would be a full “digital” switch from an H3K36me3 to H3K27me3 nucleation peak in an increasing proportion of cells as the duration of cold exposure increases [36]. The latter digital scenario has been found for the spreading of the silencing modifications across the body of the gene after transfer back to the warm [2]. Regardless of the mechanism, the opposing dynamics of H3K36me3 and H3K27me3 in the nucleation region are clearly tightly regulated by temperature. We therefore propose that this balance is used by plants to “register” the duration of cold exposure at the *FLC* locus.

## Supplemental Information

Document S1. Supplemental Experimental Procedures and Figures S1 and S2

Table S1. Primers Used in The Paper, Related to Figure 1

Document S2. Article plus Supplemental Information
