# Supplementary material for: Antagonistic Roles for H3K36me3 and H3K27me3 in the Cold-Induced Epigenetic Switch at Arabidopsis FLC
Source: Curr Biol. 2014 Aug 4;24(15):1793–7. doi: 10.1016/j.cub.2014.06.047 (PMC4123163; doi:10.1016/j.cub.2014.06.047)
Supplement: Document S1. Supplemental Experimental Procedures and Figures S1 and S2 [file mmc1.pdf]

Current Biology, Volume *24*

Supplemental Information

Antagonistic Roles for H3K36me3 and H3K27me3 in the Cold-Induced Epigenetic Switch at *Arabidopsis FLC*

Hongchun Yang, Martin Howard, and Caroline Dean

Supplemental information

Supplemental Figures and Legends

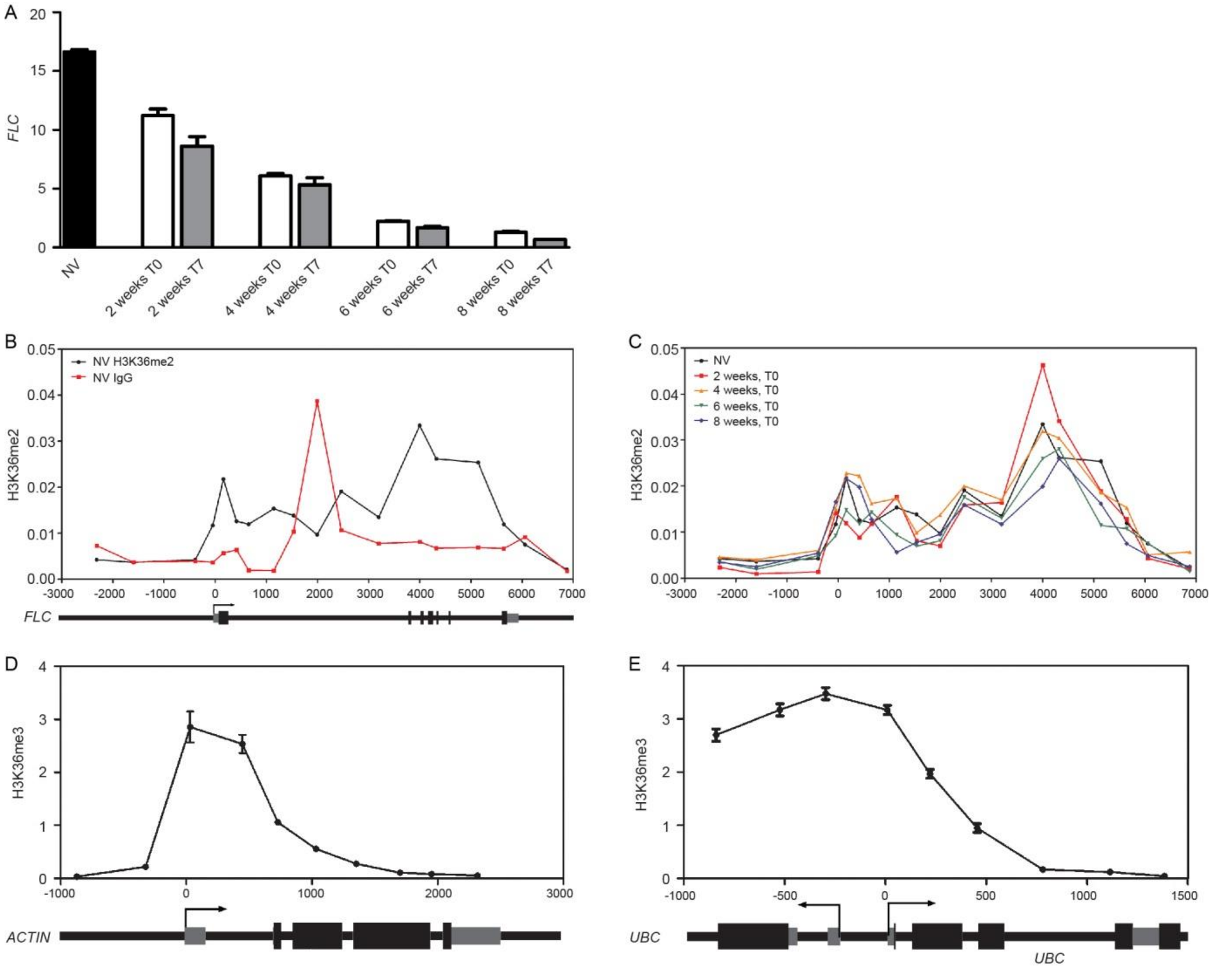

Figure S1, related to Figure 1. Characterization of chromatin modifications at *FLC*, *ACTIN* and *UBC*.

(A) *FLC* silencing in the plant material used in the ChIP experiments. The materials were harvested after the indicated treatments. Levels are relative to *UBC*. Values represent the average and SEM of three independent biological replicates.

(B) H3K36me2 levels for non-vernalized plants. The IgG ChIP was used as a negative control. Values are relative to H3 (H3K36me2 or IgG *FLC*/H3 *FLC*).

(C) Levels of H3K36me2 at *FLC* for non-vernalized plants (NV) and after 2, 4, 6 and 8 weeks of cold treatment, without post-cold growth (T0), respectively.

Values represent as H3K36me2 *FLC*/H3 *FLC* in (B) and (C).

(D) and (E) H3K36me3 patterns at *ACTIN* (D) and *UBC* (E). The schematic gene structures of *ACTIN* and *UBC* are shown. 0 is relative to transcription start site. Data were relative to H3 (H3K36me3 *ACTIN* /H3 *ACTIN* or H3K36me3*UBC*/ H3 *UBC*). Data shown are average and SEM from 3 independent biological replicates.

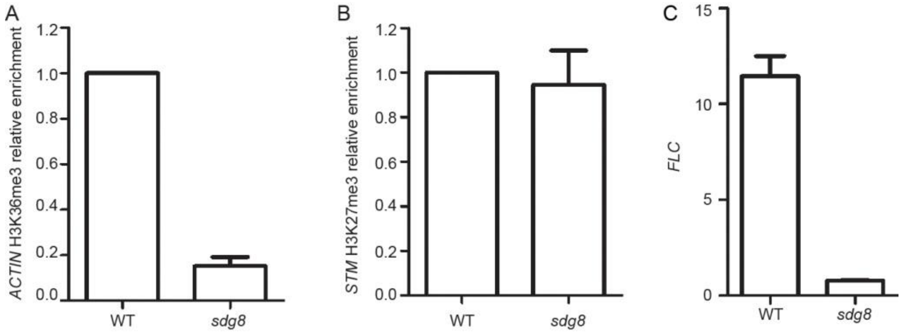

Figure S2, related to Figure 3. Characterization of *sdg8* mutant.

(A) Relative H3K36me3 levels at *ACTIN* in WT and *sdg8* mutant.

(B) Relative H3K27me3 levels at *STM* in WT and *sdg8* mutant.

Data in (A) and (B) represent the average and SEM of three independent biological replicates.

(C) *FLC* expression in *sdg8* mutant in NV conditions. *FLC* mRNA expression was determined relative to *UBC*. Values represent the average and SEM of three independent biological replicates.

Supplemental Experimental Procedures

Plant material and growth conditions

All plants used are in the *Columbia FRI sf2* background and described previously [S1]. *sdg8* was described previously [S2]. Plant growth conditions were described previously [S1]. T0 seedlings were harvested immediately after the indicated cold treatments in a cold room. T7 seedlings were grown for 7 days after transfer back to warm conditions.

ChIP and Q-PCR

Nuclei were extracted using Honda buffer as described previously [S3]. The sonication, immunoprecipitation, DNA recovery and purification were performed as previously described [S1]. The antibodies used were: anti-H3 (Abcam, ab1791), anti-H3K27me3 (Millipore, 07-449), anti-H3K36me2 (Millipore, 07-369), anti-H3K36me3 (Abcam, ab9050), anti-H3K4me3 (Millipore, 04-745), anti-H3K4me2 (Millipore, 07-030) and normal rabbit IgG (Millipore, PP64). All ChIP data were quantified by quantitative real-time PCR (Q-PCR). Values represent the average and SEM of three independent biological replicates in all cases. *FLC* expression was determined relative to *UBC*.

Western blots and immunoprecipitation

Nuclei were prepared using Honda buffer. The nuclei pellet was suspended in nuclei lysis buffer (50 mM Tris-HCl pH8.0, 10 mM EDTA pH8.0, 1% SDS, cocktail). After extreme sonication, the lysis was centrifuged at 12,000 rpm for 10 min. The supernatant was collected as the histone extract for western blots and immunoprecipitation. For immunoprecipitation, the histone extract was diluted 10 times by dilution buffer (1.1% Triton X-100, 1.2 mM EDTA pH8.0, 16.7 mM Tris-HCl pH8.0, 167 mM NaCl), then incubated with anti-H3K27me3 or anti-H3K36me3 for 3 h at 4 °C and captured by the Dynabeads protein-A (Invitrogen 10002D). The beads were washed 4 times with washing buffer (50 mM Tris-HCl

pH8.0, 2 mM EDTA pH8.0, 1% Triton X-100, 0.1% SDS, 200 mM NaCl). The immunoprecipitated histones were eluted with SDS-PAGE loading buffer.

Supplemental Reference

S1. Angel, A., Song, J., Dean, C., and Howard, M. (2011). A Polycomb-based switch underlying quantitative epigenetic memory. Nature *476*, 105-108.

S2. Ko, J.H., Mitina, I., Tamada, Y., Hyun, Y., Choi, Y., Amasino, R.M., Noh, B., and Noh, Y.S. (2010). Growth habit determination by the balance of histone methylation activities in Arabidopsis. EMBO J *29*, 3208-3215.

S3. Sun, Q., Csorba, T., Skourti-Stathaki, K., Proudfoot, N.J., and Dean, C. (2013). R-loop stabilization represses antisense transcription at the Arabidopsis FLC locus. Science *340*, 619-621.
